# Biological removal of phenol from saline wastewater using a moving bed biofilm reactor containing acclimated mixed consortia

**DOI:** 10.1186/2193-1801-3-112

**Published:** 2014-02-26

**Authors:** Seyyed Ali Akbar Nakhli, Kimia Ahmadizadeh, Mahmood Fereshtehnejad, Mohammad Hossein Rostami, Mojtaba Safari, Seyyed Mehdi Borghei

**Affiliations:** Biochemical and Bioenvironmental Research Center (BBRC), Department of Chemical and Petroleum Engineering, Sharif University of Technology, PO Box 11155-9465, Azadi Ave, Tehran, Iran; Department of Biological Sciences, Shahid Beheshti University, PO Box 19839-63113, Velenjak, Tehran, Iran; Department of Chemical Engineering, Amirkabir University of Technology, PO Box 15875-4413, Tehran, Iran

**Keywords:** Saline wastewater, Phenol, Biological treatment, Inhibitory effect, Acclimated biomass, MBBR

## Abstract

In this study, the performance of an aerobic moving bed biofilm reactor (MBBR) was assessed for the removal of phenol as the sole substrate from saline wastewater. The effect of several parameters namely inlet phenol concentration (200–1200 mg/L), hydraulic retention time (8–24 h), inlet salt content (10–70 g/L), phenol shock loading, hydraulic shock loading and salt shock loading on the performance of the 10 L MBBR inoculated with a mixed culture of active biomass gradually acclimated to phenol and salt were evaluated in terms of phenol and chemical oxygen demand (COD) removal efficiencies. The results indicated that phenol and COD removal efficiencies are affected by HRT, phenol and salt concentration in the bioreactor saline feed. The MBBR could remove up to 99% of phenol and COD from the feed saline wastewater at inlet phenol concentrations up to 800 mg/L, HRT of 18 h and inlet salt contents up to 40 g/L. The reactor could also resist strong shock loads. Furthermore, measuring biological quantitative parameters indicated that the biofilm plays a main role in phenol removal. Overall, the results of this investigation revealed that the developed MBBR system with high concentration of the active mixed biomass can play a prominent role in order to treat saline wastewaters containing phenol in industrial applications as a very efficient and flexible technology.

## Introduction

Several industries including olive oil mills, pickled vegetables, fish processing, meat canning, dairy products, tanning process and oil refining process generate wastewaters containing high salt content and high organic concentration (Lefebvre and Moletta 2006). Phenol and other phenolic compounds are common organic contaminants found in saline wastewaters formed by some of these industries such as olive oil mills, tannery and oil refinery, ranging from one to several hundred milligrams per liter (Moussavi et al. 2009; Edalatmanesh et al. 2008; Chiaiese et al. 2011).

Phenol has been classified as a priority hazardous organic pollutant regulated by the American Environmental Protection Agency (EPA) (Aravindhan et al. 2009). Thus, in order to protect human health and ecosystems from the potential toxic effects caused by exposure to phenol, its removal from saline wastewater with an efficient and environmentally benign technology is quite obligatory (Pan and Kurumada 2008). Busca et al. (2008) have recently published a short review of different technologies for phenol removal from wastewater, including physical, chemical and biological processes. However, despite its importance, few studies have been accomplished on phenol removal from saline wastewaters.

Biological processes have advantages to physico-chemical processes in pollution control due to their ability to efficiently degrade the pollutants in an environmentally sound and cost effective way (Karthik et al. 2008) and offer efficient removal of wide range of pollutants in wastewater treatment. Although wastewaters containing high-concentrations of phenol are generally difficult to treat biologically due to substrate inhibition (Ho et al. 2009); the efficient biodegradation of phenol can be obtained by microbial acclimation. Phenol can be degraded by pure cultures as well as mixed bacterial consortia (Bajaj et al. 2008). In addition, biological treatment of saline wastewater usually results in low removal efficiencies because of the adverse effects of salt on microbial flora (Uygur 2006), but by a proper adaptation of the biomass to a desired salt concentration or use of halophilic microorganisms, the detrimental effects of salinity on the overall bioprocess performance can be also mitigated (Aloui et al. 2009; Moussavi et al. 2010). Among the bioprocesses invented for biological treatment of wastewater, bioreactors implying biofilm systems play important roles in the detoxification of hazardous organic contaminants such as phenol (Hosseini and Borghei 2005). Moving bed biofilm reactor (MBBR) is a highly effective biological treatment process that was introduced about 30 years ago and now it is used in large-scale all over the world (Rusten et al. 2006). MBBR is a completely mixed and continuously operated biofilm reactor that is designed to offer the positive aspects of biofilm process including a stable removal efficiency of toxic pollutants, compact and simplicity of operation; without its drawbacks including high head loss, medium channeling and clogging (Chen et al. 2008; Delnavaz et al. 2010). In addition, moving bed reactors provide a better control of biofilm thickness and higher mass transfer characteristics (Moussavi et al. 2009). The concentration of biomass in MBBR can be increased either by raising the amount of moving media (Bassin et al. 2011), or using media with a high effective biofilm surface area, that enhances resistance to toxicity and consequently improves MBBR performance. As a consequence of such advantages MBBR process has been recently used for the removal of many toxic wastewaters including landfill leachate (Chen et al. 2008), aniline (Delnavaz et al. 2010), ammonium from saline wastewater (Bassin et al. 2011), coal gasification wastewater (Li et al. 2011), thiocyanate (Jeong and Chung 2006) and antibiotic fermentation-based pharmaceutical wastewater (Xing et al. 2013).

Treatment of phenol-laden saline wastewater using MBBR has not been reported yet. Reported applications that have dealt with biological process are mostly limited to the cases of single microbial species or low inlet phenol concentrations (Dosta et al. 2011; Afzal et al. 2007; Leitão et al. 2007; Kobayashi et al. 2007); both of which may have limitations in the field application that contaminant concentrations of targeted wastewater may alter from low to high. Accordingly, the basic purpose of this research was to investigate the performance of an aerobic MBBR to examine the above-mentioned benefits in treating synthetic phenol-laden saline wastewater using a mixed culture that gradually acclimated to phenol and salt. To achieve this aim, the MBBR was operated at different operational conditions including inlet phenol concentration, hydraulic retention time (HRT), inlet salt content and shock loadings.

## Materials and methods

### MBBR experimental setup

**Figure 1:**
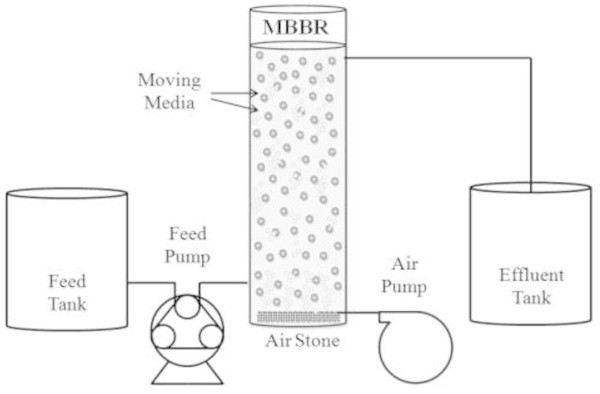
**Flow diagram of MBBR experimental setup.**

The study was performed using the cylindrical MBBR reactor (see Figure 1 for more details), made from Plexiglas with internal diameter, height and wall thickness of 14, 75 and 0.5 cm respectively, equivalent to 11.5 L total volume. The effective depth of wastewater in the reactor was 65 cm (10 L working volume) filled with up to 50% the floating biofilm carrier elements composed of high density polyethylene (HDPE) with a density of 0.96 g/cm^3^ and an effective surface area of 520 m^2^/m^3^. The aeration system was proceeded with the aid of central compressed air system. Fine bubbles were produced by the aeration system. These bubbles could provide a sufficient mixing to keep the carriers moving in the reactor. In order to keep the carriers in the reactor, outlet diameter was designed to be smaller than carrier’s size. Synthetic wastewater was injected to the reactor by a peristaltic pump with a flow controlling mechanism. Pumping rate of the wastewater into the reactor was regulated according to the working volume and HRT. In order to take samples and monitor the performance of the system in phenol and chemical oxygen demand (COD) removal, two sampling ports were provided on the influent and effluent lines of the reactor.

### MBBR operation procedure

The MBBR used in this investigation was operated at continuous mode (except in biomass acclimation phase) for 251 days. Different phases of the experiment and the range of investigated variables are presented in Table 1. In continuous system, the MBBR was run to investigate the effects of different operational variables on phenol and COD removal at the steady state operational conditions; which it was assumed that the steady state condition occurred when changing in the removal efficiency was within ±5% for consecutive HRTs at each operational run. The reactor was operated at room temperature (23 ± 2 ºC) under dissolved oxygen (DO) concentration of 4–5 mg O_2_/L controlled by regulating the aeration rate.

**Table 1:** **Experimental phases and MBBR operation timing schedule**

Phase	Day	Operation	Inlet concentration, Cin (mg/L)		Salt content (g/L)	HRT (h)
			Phenol	COD		
1	0–90	Biomass acclimation	50–500	107.5–1075	0–30	-
2	91–150	Effect of C_in_	200–1200	430–2580	30	24
3	151–190	Effect of HRT	800	1720	30	8–24
4	191–245	Effect of salt content	800	1720	10–70	18
5	246–247	Response to organic shock loading	-	-	30	18
6	248–249	Response to hydraulic shock loading	800	1720	30	-
7	250–251	Response to salt shock loading	800	1720	-	18

### Wastewater and inoculum preparation

Synthetic wastewater was prepared daily by adding phenol, nutrient stock solution and NaCl to tap water. Phenol was the sole carbon and energy source for the biomass in the MBBR. The nutrient solution consisted of Urea as a nitrogen source, (NH4)3PO_4_.3H_2_O as a nitrogen and phosphorus source and the trace elements. The COD:N:P ratio in the feed wastewater was kept at 100:5:1 throughout the experiment, where 1 mg/L of phenol is equal to 2.15 mg/L of COD. All chemicals were of analytical grade except for NaCl, which was purchased commercially. In addition, the pH of the inlet wastewater was kept at neutral range.

During the start-up, the MBBR was inoculated with an activated sludge obtained from Pars oil wastewater treatment plant. The health of the activated sludge was verified by microscopic study. Acclimation of the activated sludge to phenol and salt was lasted 90 days and was performed in the batch system. First, phenol concentration in the reactor was increased step-wise up to 500 mg/L, and then salt content in the reactor was increased step-wise up to 30 g/L. Phenol concentration remained at 500 mg/L during this stage. In each phenol and salt concentration, the reactor was operated until the removal efficiency of phenol exceeded 90% after passing 1 day. During this phase, the biofilm was gradually formed on the carriers. Microscopic observations revealed that the active and enriched salt-tolerant phenol-degrading biofilm was achieved. This biomass was used as an inoculum to the reactor.

### Analytical method

To evaluate the performance of the MBBR, samples from inlet and outlet of the reactor was taken and analyzed at HRT interval. The measured parameters in inlet samples were phenol, COD, chloride, ammonia nitrogen, phosphate and pH; whereas phenol, COD and chloride were measured in outlet samples. The parameters of pH, DO and temperature of the mixed liquor were daily measured in order to control the optimum condition for bacterial growth in the reactor. For evaluating biomass characterization the parameters of mixed liquor suspended solid (MLSS), biofilm solid (BS), biofilm thickness and specific oxygen uptake rate (SOUR) were measured in the mixed liquor and the carriers samples routinely. In order to measure phenol and COD, the samples were filtered through a filter with 0.45 mm pore size before analysis. Phenol concentrations were measured spectrophotometrically, using a Unico-UV 9200 UV/VIS Spectrophotometer by the colorimetric 4-aminoantipyrine according to the procedure given in the Standard Methods (APHA 2005). The pH, DO and temperature were measured using specific electrodes. The parameters of chloride, ammonia nitrogen, phosphorous, and MLSS were determined according to the Standard Methods (APHA 2005). The Parameters of COD for saline samples, BS, biofilm thickness and SOUR were determined according to the procedures used by Vyrides and Stuckey (2009); Plattes et al. (2006); Horn et al. (2003) and Moussavi et al. (2009), respectively. The morphology of the biomass was visualized using a microscope with 1000× magnification factor.

## Results and discussion

### Effect of inlet phenol concentration on removal efficiency

**Figure 2:**
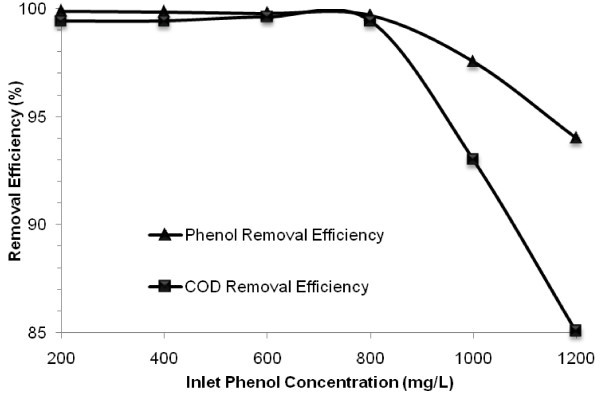
**Phenol and COD removal efficiencies versus inlet phenol concentrations at HRT of 24 h and salt content of 30 g/L.**

The effect of inlet phenol concentration ranging from 200 to 1200 mg/L (430–2580 mg COD/L) on the performance of the MBBR in phenol and COD removal efficiency was assessed over six runs under the conditions given in Table 1. Figure 2 depicts average phenol and COD removal efficiency resulted from defined steady-state conditions versus inlet phenol concentration.

As demonstrated in Figure 2 increasing inlet concentration up to 800 mg/L did not significantly affect the performance of the MBBR in phenol and COD removal and the efficiencies were over 99% for both parameters. This denotes that the part of phenol metabolized as a carbon and energy source has been completely biodegraded, although further increasing inlet concentration showed an adverse effect on the removal efficiency. Particularly, increasing inlet concentration to 1000 and 1200 mg/L resulted in decreasing phenol removal below 97.6% and 94%, respectively. Rate of decreasing COD removal was higher than that of phenol and was below 93% and 85.1% at inlet concentration of 1000 and 1200 mg/L, respectively.

The results might be explained by consideration that at low phenol concentrations no effect is noted on gross measures of metabolic activity such as specific growth rate, respiration rate, rate of synthesis, etc. By increasing phenol concentration, the biological parameters will increase due to stimulation of metabolism of the microorganisms. Eventually, the concentration is reached to a point which further increase of the concentration does not increase the biological parameters. Further increasing phenol concentration will eventually cause the physiological parameters decrease and the substrate utilization inhibition to occur (Hosseini and Borghei 2005).

Because phenol was the sole substrate the difference between COD equivalent of measured phenol and COD measured in the effluent could be explained by accumulation of organic intermediates (metabolites) that were generated during the partially phenol biodegradation caused by inhibitory effect of high phenol concentration in synthetic saline wastewater on microbial activities (Moussavi et al. 2010). The concentration of metabolites at inlet concentration of 1000 and 1200 mg/L was 97.3 and 230.6 mg/L as COD, respectively.

The results revealed that at phenol concentrations less than 800 mg/L, a complete mineralization occurred and no metabolites were generated under the given conditions of operation. Thus, this value was selected as an optimum inlet phenol concentration for the following phases of the experiment. Therefore the optimum surface loading rate based on inlet concentration (at HRT of 24 h) on the MBBR was found to be 3.08 g phenol/m^2^.day (6.62 g COD/m^2^.day). Accordingly, the MBBR could effectively remove both phenol and its COD from the synthetic saline wastewater.

These kinds of behavior and conclusions have also been shown by other researchers, although by using a pure culture (Afzal et al. 2007; Leitão et al. 2007; Kobayashi et al. 2007) or a phenol degrading mixed culture (Moussavi et al. 2010) that cannot be applicable in industrial scale. High capacity of the investigated MBBR to complete removal of phenol in saline wastewater could be attributed to use of the mixed culture of gradually acclimated active biomass to phenol and salt, using the biofilm carriers with high specific surface area available for microbial growth and high filling ratio.

### Effect of hydraulic retention time on removal efficiency

**Figure 3:**
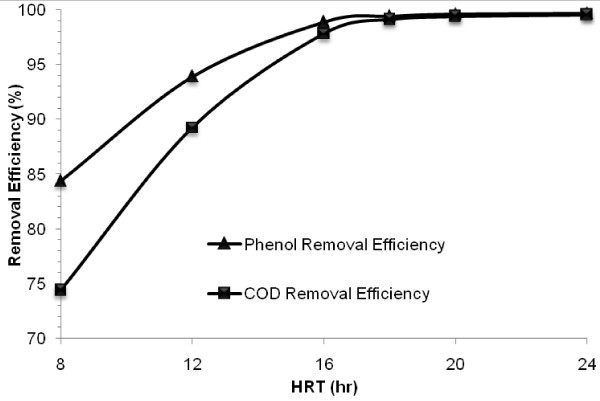
**Profile of phenol and COD removal efficiencies versus HRT at optimum phenol concentration of 800 mg/L and salt content of 30 g/L.**

In order to determine the required retention time for the efficient removal, which specifies the size of facilities in biological wastewater processes, the next phase of the experiment was designed to assess the effect of various HRT of 24, 20, 18, 16, 12 and 8 h on the performance of the MBBR in phenol and COD removal under the operating conditions given in Table 1. The reactor was operated during each HRT until defined steady-state conditions were attained. The mean phenol and COD removal efficiencies versus HRT are demonstrated in Figure 3.

Figure 3 shows that phenol and COD removal efficiencies were not affected by reducing HRT down to 18 h and the removal efficiencies of both parameters were greater than 99%. Although by further reducing HRT, the removal efficiencies of both phenol and COD were reduced and at higher values of HRT, the investigated MBBR was less sensitive to reduction of HRT. By reducing HRT to 16, 12 and 8 h, the mean removal efficiency of phenol decreased to 98.9%, 93.9% and 84.4%, respectively. COD removal efficiency decreased with higher rate in comparison to that of phenol and at HRT of 16, 12 and 8 h the mean removal efficiency of COD was below 97.8%, 89.2% and 74.4%, respectively.

This behavior might be explained by considering that the decrease of HRT until the retention time is enough for complete oxidation, has no remarkable effect on the removal efficiency. Further decreasing HRT leads to incomplete degradation. In addition, increase in hydraulic load speeds leads to detachment of the biofilm from the carrier elements and reduction of active biomass in the reactor (Hosseini and Borghei 2005).

Reducing HRT and consequently increasing phenol loading rate over the biodegradation capacity of the biomass in the reactor might inhibit the complete mineralization resulted in increasing metabolites concentration in the effluent. There was no considerable accumulation of metabolites down to retention time of 18 h. By reducing HRT to16, 12 and 8 h, phenol inhibition occurred and metabolites concentration in the effluent was increased to 18.8, 80 and 171.2 mg COD/L, respectively.

It can be concluded from above that an optimum HRT for the MBBR under the selected operational conditions was 18 h, at which phenol and COD removal efficiencies were above 99% and no metabolites were detected. Thus, this value was selected as the optimum retention time for the next phases of the experiment. Accordingly, the optimum surface loading rate based on HRT (at inlet phenol concentration of 800 mg/L) on the MBBR was found to be 4.1 g phenol/m^2^.day (8.82 g COD/m^2^.day). These results indicate that the MBBR inoculated with the active mixed biomass adapted to phenol and salt can efficiently remove high phenol loading rate and associated COD.

The adverse effect of HRT on COD removal efficiency in the MBBR system has also reported by other researchers (Li et al. 2011; Hosseini and Borghei 2005). Based on the available literature, no experiments were found dealing with removal of phenol from saline wastewater by using mixed active cultures adapted to phenol and salt in the MBBR. In comparison to other bioreactors, the investigated MBBR indicated a high performance in the removal of phenol and COD from saline wastewater. Moussavi et al. (2010) worked on phenol removal from saline wastewater with a granular sequencing batch reactor (GSBR) containing phenol-degrading consortia adapted to salt under operational conditions of cycle time of 17 h and inlet phenol concentration of 1000 mg/L, finding 99% removal efficiency. Dosta et al. (2011) evaluated the performance of a membrane biological reactor (MBR) for removing phenol from saline wastewater at HRT of 12–17 h, inlet phenol concentration of 8–15 mg/L and reported COD removal efficiency of over 98.5%. The great performance of the MBBR in this study could be especially due to the existence of a high concentration of the acclimated and active mixed culture of biomass and using a high percentage occupation of the carriers with a high effective surface area.

### Effect of salt content on removal efficiency

**Figure 4:**
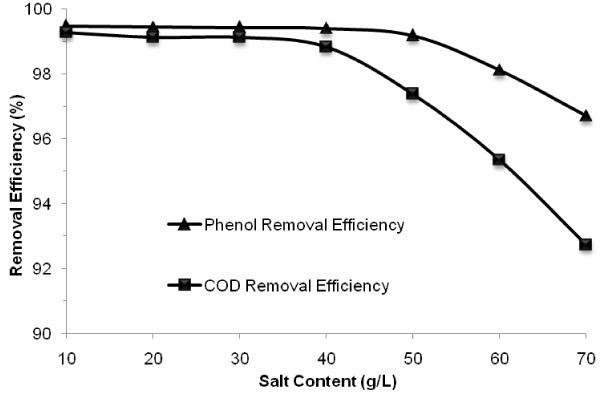
**Average of phenol and COD removal efficiencies versus inlet salt content at optimum condition of phenol concentration and HRT.**

In this phase of the experiment, the effect of salt content of synthetic saline wastewater ranging from 10 g/L to 70 g/L was assessed on the behavior of the MBBR under the previously optimized conditions given in Table 1. The MBBR was operated at each salt concentration until determined steady-state condition was achieved. The mean phenol and COD removal efficiencies as a function of salt concentration in the feed stream are demonstrated in Figure 4.

According to Figure 4 inlet salt content in the range of 10–50 g/L had negligible effect on the performance of the MBBR in phenol removal and the efficiency remained greater than 99%. However, further increase in salt concentration to 60 g/L and subsequently to 70 g/L, resulted in decrease in phenol removal efficiency down to 98.1% and 96.7%, respectively. The effect of salt content up to 40 g/L on COD removal was insignificant and the efficiency remained around 99%. However, when salt content was increased to 50, 60 and 70 g/L, COD removal efficiency was reduced down to 97.4%, 95.3% and 92.7%, respectively.

Gradually acclimation of the biomass to specific salt concentration can mitigate the detrimental effect of salinity on microbial activity. Much more salt content causes disintegration of cells because of the loss of cellular water (plasmolysis) or the recession of the cytoplasm which is induced by an osmotic difference across the cell wall and cause of outward flow of intracellular water resulting in the loss of microbial activity and cell dehydration (Abou-Elela et al. 2010).

By decreasing microbial activity in high salt content, metabolites concentration was increased to 9.6, 31, 47.5 and 68.2 mg COD/L in salt content of 40, 50, 60 and 70 g/L, respectively.

Hinteregger and Streichsbier (1997) worked on the effect of salt content (1–14%) on biotreatment of saline phenolic wastewater by a moderately halophilic strain and showed the adverse effect of salt on biotreatment. Nonetheless, Moussavi et al. (2010) showed that salt content in the range of 3–8% has no effect on the GSBR performance containing the phenol-degraded biomass adapted to salt. Stability of the MBBR against high salt content in the range of 10–40 g/L can be related to establishing the biomass containing a high concentration of salt-adapted microorganisms. It can be inferred from above that the operating MBBR with the mixed consortia acclimated biomass can attain a high performance for phenol-laden saline wastewater in terms of phenol and COD removal under the different operational conditions.

### Response to shock loading

Shock loading can be applied by sudden increase of organic concentration, flow rate and salt content in saline wastewater. Therefore in this phase of the study, response of the investigated MBBR to mentioned shock loads was evaluated.

### Response to organic shock loading

**Figure 5:**
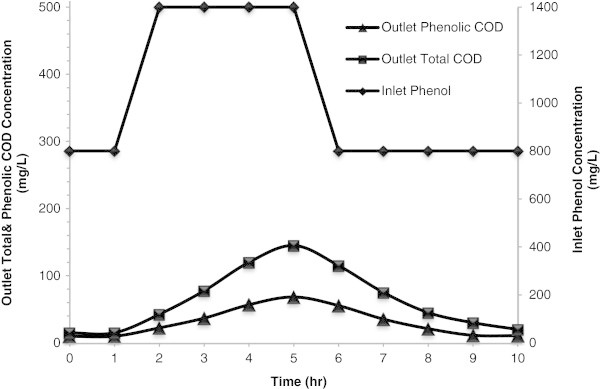
**Variation of outlet total and phenolic COD concentration during phenol shock load at the HRT of 18 h and salt content of 30 g/L.**

In order to evaluate the adverse effect of phenol shock load on the MBBR performance, a sudden increase of inlet phenol concentration from 800 to 1400 mg/L was applied to the bioreactor for a period of 4 h under the conditions presented in Table 1. During this period and after that the concentrations of outlet phenol and COD were monitored every 1 h until the steady state condition was re-established, as shown in Figure 5. It can be seen that outlet phenolic and total COD were increased from 10 and 15 mg/L to the maximum concentrations of 68.4 and 145 mg/L, respectively. Around 5 h after shock load outlet phenolic and total COD gradually reached to the near steady state values of 10.75 and 25 mg/L, respectively.

### Response to hydraulic shock loading

**Figure 6:**
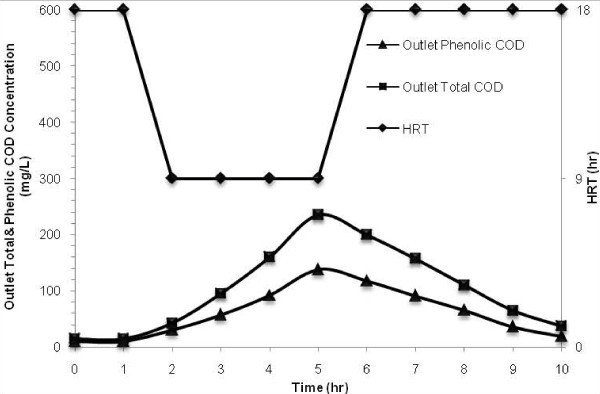
**The effect of hydraulic shock load on the performance of the MBBR at inlet phenol concentration of 800 mg/L and salt content of 30 g/L.**

In order to study the reactor stability against a sudden variation of flow rate, HRT was changed from 18 to 9 h for a period of 4 h under the conditions listed in Table 1. To understand the trend of outlet phenolic and total COD concentration during shock loading and after that, the effluent was sampled and analyzed at 1-h-intervals and the results are shown in Figure 6. According to Figure 6 outlet phenolic and total COD started to increase from 10 and 15 mg/L to the maximum concentrations of 138 and 235 mg/L, respectively; then gradually started to decrease to the near steady state value of 18.7 and 37.5 mg/L about 5 h after shock load.

### Response to salt shock loading

**Figure 7:**
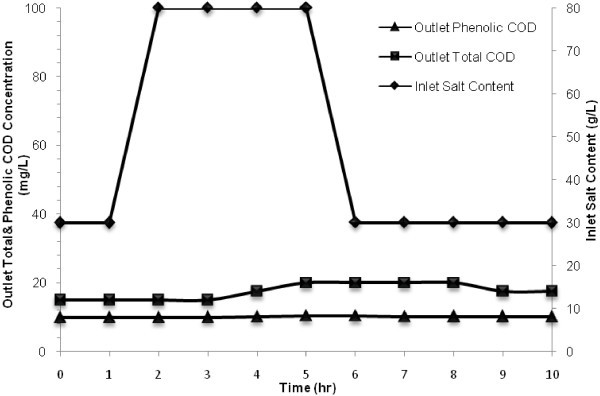
**Effect of salt shock load on outlet total and phenolic COD at inlet phenol concentration of 800 mg/L and HRT of 18 h.**

To evaluate the resistance of the MBBR against jump of inlet salt concentration, a sudden change was applied to the reactor where inlet salt concentration increased from 30 to 80 g/L for a period of 4 h under the conditions listed in Table 1. The changes in outlet phenolic and total COD concentration under this condition and after that were monitored hourly and the results are presented in Figure 7. Figure 7 depicts that outlet phenolic and total COD concentrations changed insignificantly and were increased from 10 and 15 mg/L to the maximum concentrations of 10.3 and 20 mg/L, respectively. But after switching the influent to the initial condition, the effluent was remained unchanged, which could be because of remaining a high salt content in the bioreactor.

It can be inferred from the above results that the MBBR exhibited a high stability against organic, hydraulic and salt shock loadings and recovered from these changes in a relatively short time. This low sensitivity to shock loadings could be due to the existence of a high concentration of biomass containing gradually acclimated and active microbial consortia and a high filling ratio of the biofilm carriers with a high effective surface area in the reactor. High stability of the MBBR against shock loadings had previously reported by other researchers (Chen et al. 2008; Hosseini and Borghei 2005). These advantages introduce the MBBR as an effective and stable process for the removal of phenol from saline wastewaters.

### Biomass characteristics

The biomass characteristics were evaluated both in suspension and biofilm during this study. The determined characteristics were MLSS, BS, biofilm thickness and SOUR. The range of measured biological parameters during this study is presented in Table 2. In the continuous system, the suspended biomass in the bioreactor was negligible in comparison to the biofilm attached to the carriers. It can be inferred that the attached biomass had the main role in the removal of phenol and COD rather than the suspended biomass in the investigated MBBR. The thickness of the biofilm formed on the media was in the range of effective biofilm thickness (the depth of the biofilm to which the substrates have penetrated) (Rusten et al. 2006). The SOUR values indicate a high activity of the biomass in the reactor which could be attributed to the moving media containing a thin biofilm that improves the oxygen and substrate transfer rate and contact between the substrate and the biomass, therefore, enhances degradation rate (Moussavi et al. 2009).

**Table 2:** **Characteristics of biomass during study**

Parameter	Unit	Value
Mixed liquor suspended solid	mg/L	250–640
Biofilm solid	mg/L	1405–4450
Biofilm thickness	μm	18–58
Specific oxygen uptake rate	mg O_2_/mg VSS.d	0.62 ± 0.07

**Figure 8:**
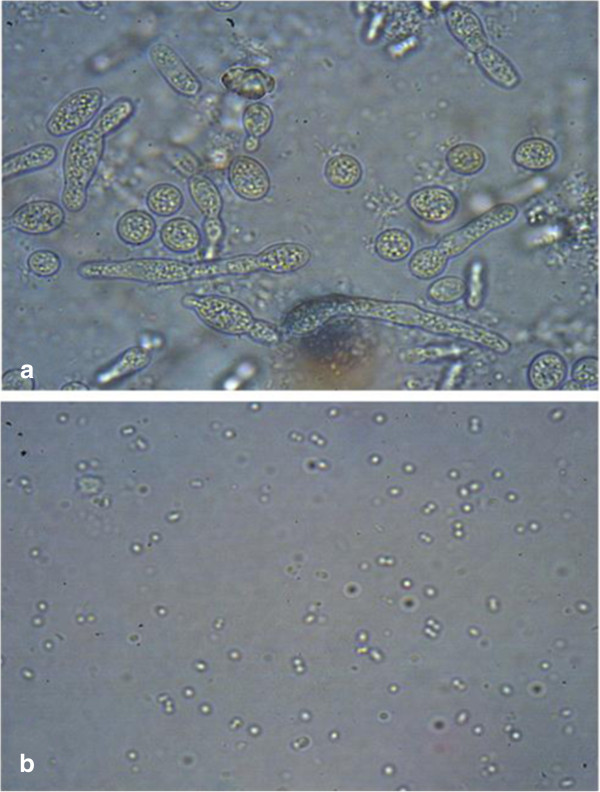
**Photomicrograph of biomass.** **(a)** biofilm **(b)** mixed liquor.

Microscopic examinations were carried out in order to observe the existing microorganisms in the bioreactor during the experiments. Photomicrograph of the biofilm and the mixed liquor are demonstrated in Figure 8. As shown in Figure 8(a) predominant species in the biofilm was yeast and some mold and bacteria also were found in the biofilm, but there was no indication of these groups of microorganisms in the liquid bulk where bacteria was the main, as shown in Figure 8(B). As demonstrated in Figure 8(A), the existence of yeast ongoing to budding and fission implied a high activity of the biofilm inside of the bioreactor. Dan et al. (2003) indicated that yeast culture is more efficient in treating high organic–high salinity wastewater compared to bacterial cultures. Hence, the high performance of the investigated MBBR in phenol removal efficiency could be attributed to the existence of the high concentration of yeast in the biofilm.

## Conclusion

The present work investigated the performance of a bench scale MBBR for phenol removal from saline wastewater. The results revealed that the MBBR provides improved phenol and COD removal efficiencies. Inlet phenol concentrations up to 800 mg/L did not significantly affect the performance of the MBBR with HRT of 24 h and salt content of 30 g/L, where phenol and COD removal efficiencies were above 99%. Optimum HRT for the reactor was 18 h, such that decreasing HRT below this value led to reduction of the removal efficiencies of both phenol and COD. The MBBR exhibited low sensitivity to increasing salt concentrations up to 40 g/L. The reactor was very stable against phenol, hydraulic and salt shock loadings and performed well under various operational conditions. The active biofilm containing yeast as a predominant species performed the main role in phenol removal in the MBBR. Overall, high performance of the investigated MBBR in the removal of phenol from saline wastewater could be attributed to existence of the mixed culture of gradually acclimated biomass to phenol and salt and using a high filling ratio of the biofilm carriers with a high effective surface area.

## Abbreviations

MBBR: Moving bed biofilm reactor
COD: Chemical oxygen demand
HRT: Hydraulic retention time
EPA: Environmental protection agency
HDPE: High density polyethylene
DO: Dissolved oxygen
MLSS: Mixed liquor suspended solid
BS: Biofilm solid
SOUR: Specific oxygen uptake rate
GSBR: Granular sequencing batch reactor
MBR: Membrane biological reactor.
